# Constructing a finer-grained representation of clinical trial results from ClinicalTrials.gov

**DOI:** 10.1038/s41597-023-02869-7

**Published:** 2024-01-06

**Authors:** Xuanyu Shi, Jian Du

**Affiliations:** 1 Institute of Medical Technology, Peking University, Beijing, 100191 China; 2 National Institute of Health Data Science, Peking University, Beijing, 100191 China

**Keywords:** Outcomes research, Drug development, Data publication and archiving, Statistical methods

## Abstract

Randomized controlled trials are essential for evaluating clinical interventions; however, selective reporting and publication bias in medical journals have undermined the integrity of the clinical evidence system. ClinicalTrials.gov serves as a valuable and complementary repository, yet synthesizing information from it remains challenging. This study introduces a curated dataset that extends beyond the traditional PICO framework. It links efficacy with safety results at the experimental arm group level within each trial, and connects them across all trials through a knowledge graph. This novel representation effectively bridges the gap between generally described searchable information and specifically detailed yet underutilized reported results, and promotes a dual-faceted understanding of interventional effects. Adhering to the “calculate once, use many times” principle, the structured dataset will enhance the reuse and interpretation of ClinicalTrials.gov results data. It aims to facilitate more systematic evidence synthesis and health technology assessment, by incorporating both positive and negative results, distinguishing biomarkers, patient-reported outcomes, and clinical endpoints, while also balancing both efficacy and safety outcomes for a given medical intervention.

## Background & Summary

Clinical trials, specifically randomized controlled trials (RCTs), are a type of prospective, randomly allocated, blinded clinical study. RCT is the golden standard to evaluate the efficacy and safety of a single or multiple clinical interventions. In the new evidence-based medicine pyramid, RCTs are rated as the highest position, representing the most reliable medical evidence^1^.

Clinical trial results published in medical journals often exhibit significant publication bias and selective reporting^2^. Clinical researchers tend to favor positive outcomes while neglecting negative results, thereby contributing substantially to an unhealthy clinical evidence ecosystem and presenting significant challenges to high-quality, unbiased clinical decision-making. In complement to published clinical trial results, ClinicalTrials.gov (CT.gov) has been proven to be a valuable but underutilized database for clinical trial results. The unique value inherent in CT.gov data has been demonstrated through various comparisons and analyses. When compared with results from PubMed, it has been noted that CT.gov often contains a more comprehensive report of adverse events. In CT.gov, safety results were reported at a similar rate as in peer-reviewed literatures, yet with more thorough reports of certain safety events^3^. Most recently, a step-by-step guidance has been published to instruct clinical researchers on how to conduct systematic searches for registered studies as well as reported results using clinical trials registers^4^. However, the current storage of CT.gov reported results is limited to web-based or raw XML format. The absence of automated processing tools and a lack of structured reporting results dataset constitute one of the major barriers to the widespread utilization of CT.gov.

Our work aims to fill the gap. We aim to facilitate the reuse of the current under-utilized reported results dataset hidden in registries, making it more feasible for inclusion in evidence synthesis practices. In addition, parsing CT.gov reported results into a structured dataset is not only an attempt to respond to the call for computable evidence synthesis^5^, but also aligns with the recent introduced principle of “calculate once, use many times”^6^, where the clinical trial analysis results are respected as data.

Despite a study suggested that CT.gov had not altered the conclusions of systematic reviews^7^, we argue that the limited sample size in their analysis may have influenced these findings. Results of RCT, are either published in scientific articles, or reported on registry platforms, such as CT.gov, International Clinical Trials Registry Platform (ICTRP), and Chinese Clinical Trial Registry (ChiCTR). Researchers found that in the 91 trials with reported results on CT.gov and published in high-impact journals, only 52% primary efficacy end points were described in both sources and reported concordant results^8^. One of the possible and biggest explanation for this phenomenon is selective publication or publication bias of clinical trials, meaning the existence of a higher reporting rate of positive results in published literature compared to that in registries^9^.

This study intends to construct a finer-grained representation of arm-centered clinical trial results by integrating efficacy and safety information. Our methodology enables: 1) a detailed representation of “intervention” entities, transcending the traditional PICO (Population, Intervention, Comparator, Outcome) concept representation; 2) a systematic unveiling of positive and negative results in efficacy outcomes; 3) a systematic disclosure of serious adverse events (SAE) in safety outcomes; 4) a dual-faceted understanding of intervention effects from both efficacy and safety perspectives. 5) By providing a structured, ready-to-use dataset, we aspire to offer a new data source for meta-analysis, thereby facilitating a more discoverable dataset to enhance evidence-based health decision-making.

The discrepancy between PICO information in the design phase and reported results underscores the necessity for real meta-analysis which mandates results-oriented information. Our study seeks to bridge this gap, fostering a more thorough and reliable synthesis of evidence that can potentially elevate the standards of medical research and practice.

### Existing studies

There have been studies focusing on the structuring trial results data. MedicineMaps^10^ introduced a schema that represents clinical trial results from literature. The schema lost information about the comparators, and was annotated manually. EvidenceMap^11^ extracted PICO with observation data and efficacy relationships from 80 COVID-19 clinical trial abstracts. Similarly, entities such as ‘two groups’ were also labeled as interventional, losing the ability to build comparable relationships. TrialStreamer^12^ and TrialSummarizer^13^ are tools that extracts results from clinical trials in a large scale. For data from registry platform, CTKG^14^ is a large knowledge graph version with embedding analysis of studies in the CT.gov database. The knowledge graph follows the basic structure of ClinicalTrials.gov, with nodes being sections and edges being ‘has’, focusing on processing the metadata of clinical trials. The standardization of metadata and use of knowledge graph contributed to the obstacles to the reuse of study metadata in clinical trials^15^. In ‘outcome_analysis’, same as the representation in CT.gov, the compared arm groups were not separated as intervention versus comparator as well.

### In this study

We focus on results data and introduce an arm-level representation of efficacy and safety results in studies registered on CT.gov. We optimize the data structure of clinical trials and present study results with one-to-one comparable relationships. Each efficacy result is represented by a relationship from an intervention arm group to an outcome, with the efficacy as the relationship value, the comparator arm as an attribute. Each safety result is represented by a relationship from an arm group to an adverse event, with the number of affected subjects as an attribute.

**Fig. 1:**
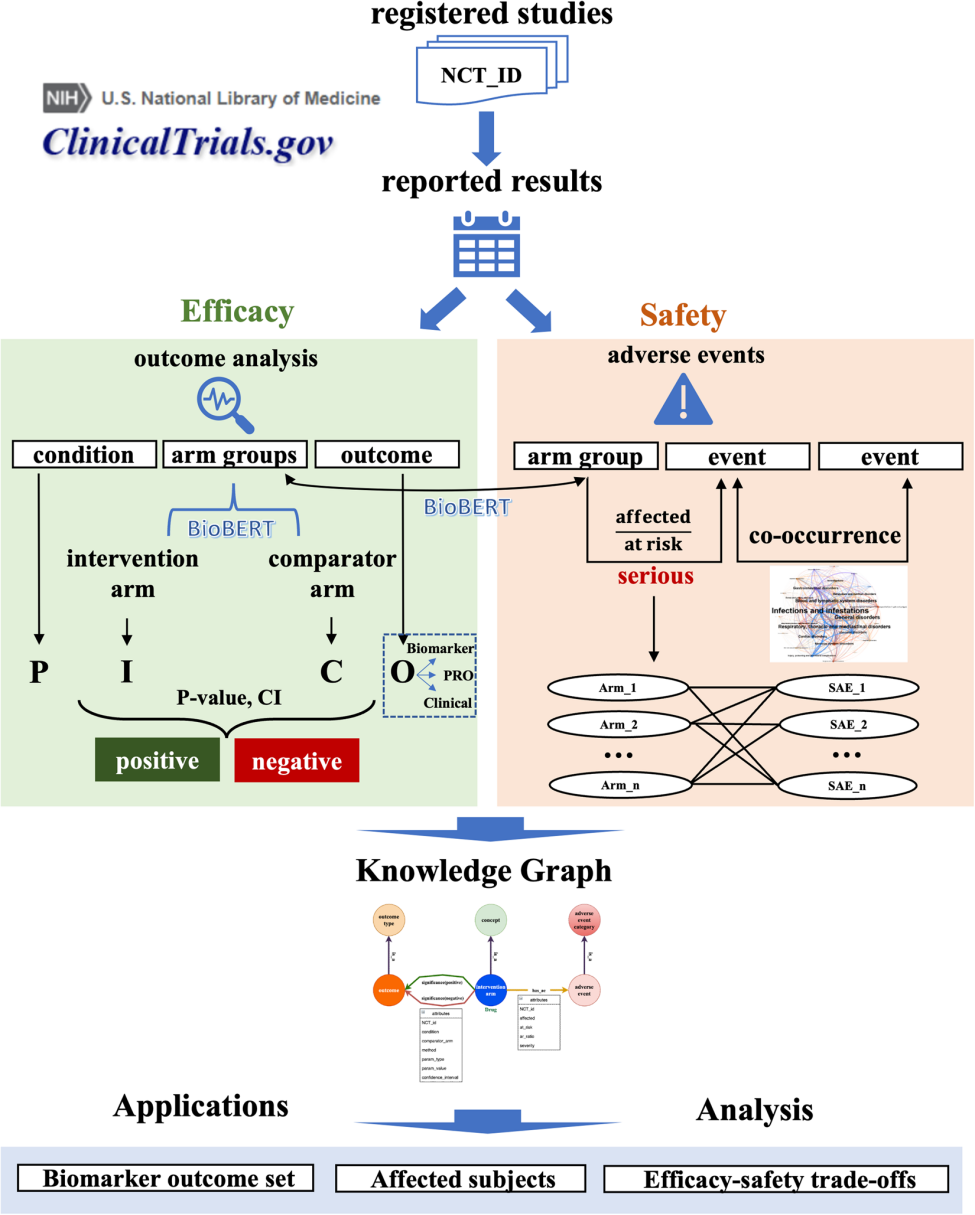
The overall workflow from data acquisition, results processing, to the final knowledge graph construction.

To represent arm-level efficacy and safety results in a knowledge graph, we started with acquiring registered clinical trials from the registry platform CT.gov, followed by extraction of statistical significance and adverse events from reported results. After separating arm groups and connecting outcomes, we eventually constructed a knowledge graph including results nodes, relationships and related attributes. Figure 1 shows the overall workflow of this study.

## Methods

### Data source

On December 25^th^ in 2022, we downloaded full registered clinical trials dataset directly from CT.gov (https://classic.clinicaltrials.gov/AllPublicXML.zip). The dataset is a compressed zip file containing all the individual raw XML files of each study named by the NCT id. The total number of XML files of clinical trials downloaded is 437,173. We parsed and transformed each raw file into a Pandas DataFrame. A Pandas DataFrame is a data structure in Python’s Pandas library, primarily used for data manipulation and analysis (https://pandas.pydata.org/docs/reference/api/pandas.DataFrame.html). Using Pandas DataFrames over individual XML files simplifies the integration and analysis of clinical trial results. This makes it easier to handle large datasets, perform complex analyses, and draw insights from clinical trial results. From the reported results section, we focus on efficacy and safety related to the intervention arm group, which are stored under the statistical analysis and adverse events section.

### Arm group-level results

In the context of clinical trials, an “arm” refers to a group of subjects receiving a particular intervention, treatment, or control. The term is often used in RCTs, which are considered the gold standard for evaluating the efficacy of treatments. In a typical RCT, participants are randomly assigned to different arms of the study to minimize the influence of confounding variables and to allow for a fair comparison of the interventions being tested.

#### Efficacy results

In evidence-based medicine, a record of clinical trial results has to be described in PICO framework, along with the efficacy (E). In clinical trials, population describes the characteristics of the selection of study subjects, such as age, gender, condition; intervention represents the treatment or strategy being studied, which can be a drug, a lifestyle or a dietary plan; comparator represents the strategy for comparison with the intervention, which can a placebo method or a standard care; outcome describes what is being measure in the study to assess the efficacy and safety of the intervention, which can be symptom relief, biomedical markers, or mortality rate.

The PICO framework is essential for formulating research questions and reviewing literatures for relevant clinical studies. In the search for the efficacy of an intervention, it is essential to determine the anticipated corresponding population, comparator, and outcome. In natural language, a clinical trial result can be expressed as: ‘There exists a significant difference between the intervention (I) and the comparator (C) on the outcome (O) in the population (P).

**Fig. 2:**
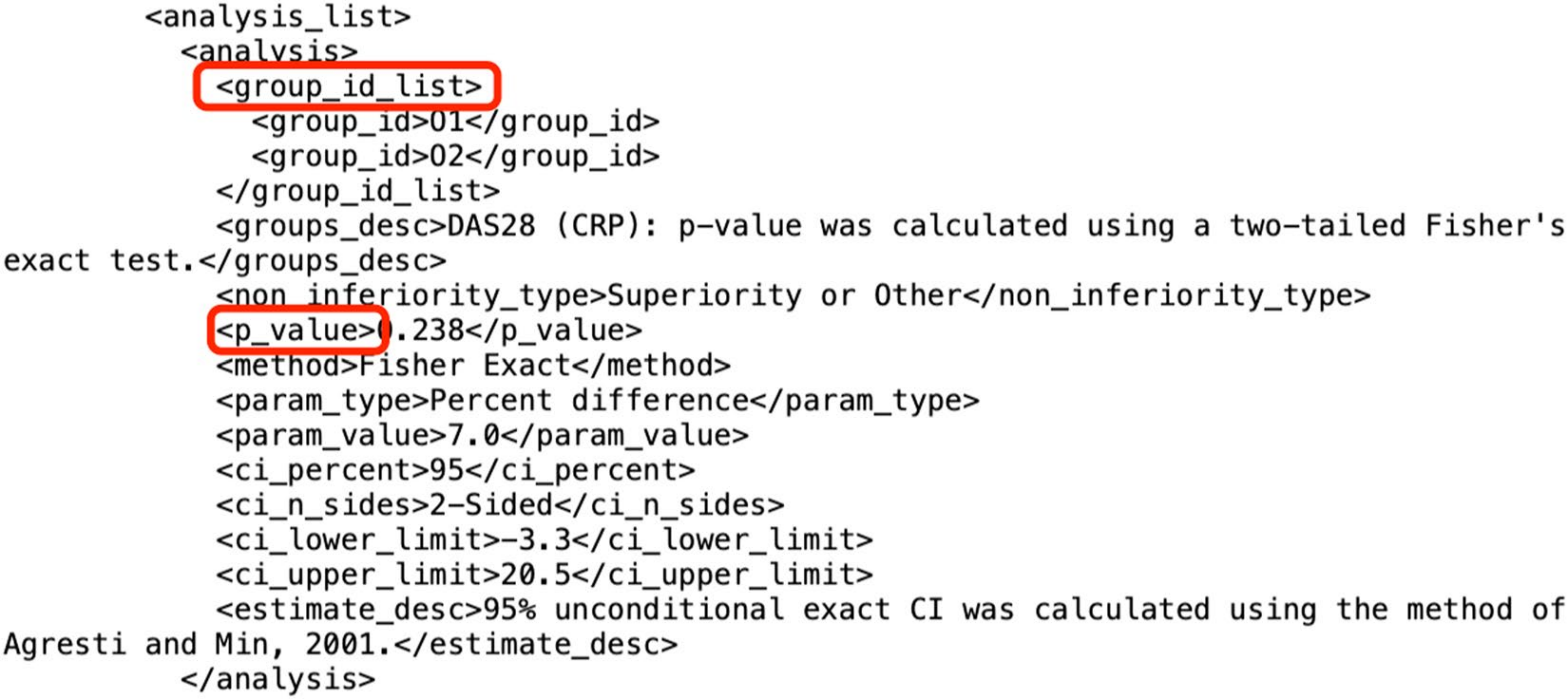
An example of statistical analysis in XML file.

In this study, to evaluate the efficacy of an intervention on an outcome, we searched for the statistical analysis section under each outcome in the study results page. For example, in the study *NCT01050998*, under the outcome measure of the primary outcome ‘*Percentage of Participants Who Achieved Disease Activity Score of 28 Joints Using C-Reactive Protein (DAS28 [CRP]) Response at Day 85 by Region’* exists a list of statistical analysis results. As we can see from an example of results (Fig. 2), the analysis section provides information such as groups, p_value, and confidence interval, etc. With these pieces of information, we are able to create a relationship between an intervention arm group and the primary outcome.

To parse the XML files for efficacy results, we extracted results in the form of relationships between ‘groups’ under each outcome measure and corresponding ‘outcome’: group-outcome. Each group-outcome pair is saved in a row in a Pandas Dataframe, along with other columns including other important attributes such as NCT_ID, p-value, statistical method, and confidence interval. An example is shown in Table 1.

**Table 1:** An example of transformed dataframe of efficacy results.

NCT_ID	Groups	Outcome_title	P_value
NCT01049373	[‘Lymphdiaral Basistropfen (HDC)’, ‘Placebo Solution’]	Change in FFbH-R Between Screening and 2 Weeks	0.0757
NCT03400800	[‘Inclisiran’, ‘Placebo’]	Percentage Change in LDL-C From Baseline to Day 510	<0.0001
NCT02912650	[‘Placebo’, ‘Ibuprofen 250 mg’]	Pain Intensity Difference on 11-Point Numerical Scale (PID11)	<0.001
NCT02954354	[‘Baloxavir’, ‘Oseltamivir’]	Percentage of Participants Reporting Normal Temperature at Each Time Point in Adults Randomized to Baloxavir or Oseltamivir	0.5908
NCT01795547	[‘Aripiprazole’, ‘Paliperidone’]	Change From Baseline to Week 28 in the ‘Interpersonal Relations’ QLS Domain Score	0.07

#### Statistical significance

To better and directly represent the efficacy of an intervention group on an outcome, we created an automatic rule-based pipeline to summarize analysis attributes for statistical significance and eventually presented as positive vs negative.

1. check if the result has valid p-value
2. if no p-value found, check if the result has valid confidence interval
3. for results with p-value, we label the result as positive if it has a p-value smaller than or equal to 0.05, and negative otherwise
4. for results without p-value but with confidence interval, we check whether the statistical parameter is a ratio-type parameter (Odds Ratio, Hazard Ratio, etc.) or a difference-type parameter (Mean Difference, Risk Difference, etc.)
5. for results with a ratio-type statistical parameter, check if the number one:1 is contained between the lower and upper confidence interval limits. If 1 is not contained, label the result as positive, and negative otherwise
6. for results with a difference-type statistical parameter, check if the number zero:0 is contained between the lower and upper confidence interval limits. If 0 is not contained, label the result as positive, and negative otherwise

The detailed visualized pipeline and corresponding number of results in each step are shown in the result section (Fig. 4).

In the XML clinical trial files downloaded from the website, arm groups are labeled with ‘Experimental’ and ‘Comparator’ in the study design section. But in the statistical analysis section, the intervention arm group is not distinguished from the comparator arm group using a label. The titles of the groups are also not always consistent with the titles from the study design section, meaning it is not feasible to simply match the labels using the exact strings. Thus, in this part, we utilized a transformer model BioBERT^16^ (‘dmis-lab/biobert-v1.1’) to automatically separate intervention arm group from comparator arm group. BERT, Bidirectional Encoder Representations from Transformers, is a transformers-based deep learning model that was introduced by Google in 2018^17^. It is a language model that is pretrained on a large corpus of texts including Wikipedia. The model allows people to invoke weights (embeddings) of texts from the pretrained model without training on their own. BioBERT is a domain-specific adaptation of the original BERT and pretrained on a large corpus on biomedical texts. With BioBERT, we are able to perform various tasks such as named entity recognition and relation extraction on biomedical problems. In our specific task, we used the version BioBERT-Base v1.1 that is trained on around one million biomedical publications on PubMed.

In this experiment, to prepare the data in the form of the knowledge graph schema, we only kept results with exact two arm groups for comparison between intervention and comparator. Studies without valid labels of ‘experimental’ and ‘comparator’ arm groups from the study design section were excluded.

We first produced semantic embeddings of the arm group titles from both the study design and statistical analysis sections using BioBERT. Then we calculated the similarities of semantic embeddings between the arm groups from different sections. Eventually we labeled the arm group from the statistical analysis section based on the label of the arm group that has the highest similarity score from the study design section. For examples shown in Table 2, the ‘Groups’ column are the original stored groups information from CT.gov. After comparing to columns ‘Intervention’ and ‘Comparator’ from the study design section, new columns ‘Intervention_group’ and ‘Comparator_group’ were created to represent the experimental and comparator groups. Validation on the applied approach is provided in the Technical Validation section.

**Table 2:** Example results of separation of arm groups into intervention vs. comparator in efficacy results.

NCT_ID	Groups	Intervention	Comparator	Intervention_group	Comparator_group
NCT03064438	[ACU-D1 Ointment, ACU-D1 Ointment Vehicle]	[ACCU-D1]	[Vehicle]	ACU-D1 Ointment	ACU-D1 Ointment Vehicle
NCT02553317	[Caplacizumab, Placebo]	[Caplacizumab]	[Placebo]	Caplacizumab	Placebo
NCT00650806	[Placebo, Canagliflozin 50 mg]	[Canagliflozin (JNJ-28431754)]	[Placebo]	Canagliflozin 50 mg	Placebo
NCT02152605	[Placebo,UMEC/VI 62.5/25 mcg]	[UMEC/VI]	[Placebo]	UMEC/VI 62.5/25 mcg	Placebo
NCT00402246	[Remote Arm, In-Office Arm]	[Remote Management]	[In-Office Care]	Remote Arm	In-Office Arm

#### Safety results

We integrated reported adverse events from CT.gov to represent potential safety harms related to the interventions. In the trial raw XML files, an adverse event is stored under the <event> section, along with <sub_title> and <count>, recording information including the corresponding group, the number of affected subjects, and the number of subjects at risk. The vast majority of adverse event titles are standardized into the Medical Dictionary for Regulatory Activities (MedDRA^18^) by CT.gov. The parent elements that incorporate the specific events cover health categories such as ‘Cardiac disorders’, ‘Ear and labyrinth disorders’, etc. The events are also classified as serious events vs. other events, giving users the ability to choose by event severity. In this study, we focus on serious adverse events of which the number of affected subjects is none-zero.

In order to intuitively compare the efficacy and safety, we only kept serious adverse events in the studies that have valid efficacy analysis results (54.8%, 6,429 out of 11,729). On CT.gov, not all the expressions of arm groups in adverse events sections are 100% same as the ones in efficacy. In order to integrate efficacy and AE, we first tried using string matching among the arm groups. For arm groups in AE that were not string-matched to an arm group in efficacy, we utilized BioBert again to calculate the semantic similarities and return the closest match. For each adverse event title, we calculated the semantic similarities between the title and all the arm groups in the efficacy section. The closest matched arm group was saved as a unique column ‘matched_group’ in the dataframe, and we used the matched arm groups for integrating efficacy and safety. The columns for adverse events we saved in the dataframe include ‘NCT_ID’, ‘arm_title’, ‘event_title’, ‘category’, ‘serious/other’, ‘affected’, ‘at_risk’, and ‘matched__group’. Each row can be comprehended as a reported adverse event named ‘event_title’ related to the arm group named ‘matched_group’ along with the number of affected and at-risk participants (Table 3).

- **Events co-occurrence**. In extra, we calculated co-occurrence weights among reported adverse events to provide the which SAE is frequently occurred with another SAE. We determined the co-occurrence of categories of SAEs by the following considerations: if both two categories have reported ‘affected’ participants in a same trial, and multiplying the ‘affected/at_risk ratio’ from the categories  $({\sum }_{(a,b)\in S,a\ne b}c(a,b)\times (r(a)\times r(b)))$ (c:co-occurrence, r:ratio). This ratio-based co-occurrence took the affected/at_risk ratio of SAE into account, representing the probabilistic extent of events co-occurrence.

**Table 3:** Example adverse events with matched group in efficacy analysis section.

NCT_ID	Arm_title	Event_title	Category	Serious/other	Affected	At_risk	Matched_group
NCT00977080	Oral Paricalcitol in the Oral Stratum	Abdominal pain	Gastrointestinal disorders	serious	1	72	Oral Paricalcitol in the Oral Stratum
NCT02254278	IMRT 5 Weeks	Syncope	Nervous system disorders	serious	1	147	IMRT 5 Weeks
NCT00509145	Laquinimod 0.6 mg	Uterine prolapse	Reproductive system and breast disorders	serious	1	550	Laquinimod
NCT00810199	Tocilizumab + Placebo	Clostridium difficile colitis	Infections and infestations	serious	1	276	Tocilizumab + Placebo
NCT02853331	Sunitinib	Hepatocellular injury	Hepatobiliary disorders	serious	1	425	Sunitinib

### Outcome

#### MeSH extraction

In clinical trials, expression of outcomes varies across different studies. Thus, we processed all the unique outcomes using the MeSH (Medical Subject Headings) extraction tool: NLM (National Library of Medicine)’s Medical Text Indexer (MTI)^19,20^. MTI allows us to extract MeSH terms from natural languages, in our case, outcomes. MeSH represents the simplest representation of biomedical languages for tasks including document retrieval, text classification, etc. For all the MeSH terms extracted, we also recorded the parent terms along with the codings based on the tree structure of MeSH. With MeSH terms of outcomes, we are able to not only standardize outcome titles with different expressions, but also classify outcomes into different clinical categories.

#### Outcome category

In clinical trials, the selection of outcomes has critical impact on the efficacy of the intervention. A study proposed categories of outcomes as Mortality/survival, Physiological/clinical, Life impact, Resource use, and adverse events^21^. By assumption, with the same intervention, choosing biomarkers as the outcome potentially tend to produce positive results^22^. Also, biomarkers are usually used as surrogate endpoints that are easier to measure. In contrast, in most cases, clinical endpoints are more complicate to measure, and tend to produce negative results. In this study, we classified the outcome titles into three categories: Biomarker, Patient-reported outcome (PRO), and clinical endpoint.

- **Biomarker**. We took the advantage of MeSH to identiy biomarker outcomes. We first manually went through the MeSH tree to discover possible biomarker-related terms. We found that the majority of the terms we discovered (such as ‘E01.370.376.537.250-Brain Cortical Thickness’, ‘D12.776-Proteins’, and ‘D10.251-Fatty Acids’) belong to the root category D (Chemicals and Drugs) and E (Analytical, Diagnostic and Therapeutic Techniques, and Equipments). Thus, we preliminarily selected the root categories D and E as the biomarker identifier in outcomes. If any of the MeSH terms extracted from an outcome title starts with D or E, we labeled the outcome as biomarker. We excluded ‘E05.318.308.980-Surveys and Questionnaires’ as it is a PRO related MeSH term. 40,525 results with unique 14,217 biomarker outcomes were identified.
- **PRO**. Patient-reported outcomes are used to report the status of a patient’s health condition from the patient’s persepective^23^. Research identified PROs and inspected the inclusion of PROs in registered clinical trials from 2007 to 2013 and found 27% of the trials used one or more PRO measures^24^. Recently, the usage of PROs in novel artificial intelligence (AI) clinical trials was assessed by researchers (152/627 trials)^23^. Both previously-mentioned studies used PRO databases such as PROQOLID and GEM to match PROs in registered clinical trials. One of the major limits of exact text-matching for outcomes in registered clinical trials is that the writings or expressions are natural languages, meaning they can be inconsistent. In this study, we simplified the searching words and took advantage of the extracted MeSH terms. To identify PRO outcomes, we applied keyword matching on columns ‘outcome_title’ and ‘outcome_mesh’. The keywords are ‘Survey’, ‘Questionnaire’, ‘Patient Reported’, ‘Patient-Reported’, ‘Scale’, and ‘Score’. We labeled the outcome as PRO if any lowercase of the keywords was matched in the outcome string. We eventually found 33,547 results with 9,820 unique PRO outcomes.
- **Clinical endpoint**. In this study, we defined the disease-related outcomes as the clinical endpoint outcomes. The root category C from MeSH tree represents ‘Diseases’. We also added F03 (Mental Disorders) as a target disease-related category. If any of the MeSH terms extracted from an outcome title starts with ‘C’ and ‘F03’, we labeled the outcome as clinical endpoint. 23,557 results with 7,837 unique clinical endpoint outcomes were identified.

#### Biomarker outcome set

We provide a dataset of biomarker outcomes with the most positive and negative efficacy results under each health problem. The dataset is provided in the supplementary Table S1. Note that the outcomes are not standardized by concept, meaning there might be variations of same outcomes that are not summed together. Besides, this example shows the top biomarker outcomes associated with health problems. In the full dataset, outcomes should be associated with the original condition name. This dataset provides clinical researchers and practitioners surrogate endpoints for measurement when it is infeasible, unethical, or ineffective to directly evaluate clinical endpoints.

### Knowledge graph construction

A knowledge graph (KG) is a specialized graph-based data structure used for representing a collection of knowledge, entities, and their relationships. In this study, we stored the clinical trial entities (outcome, arm group, adverse event, etc.) and their relationships in a knowledge graph for visual representation and complex data retrieval queries.

We created 3 main node types: intervention_arm, outcome, and adverse_event:

- **Intervention_arm**. The intervention_arm node is interventional arm group from the statistical analysis result section. The relationship representing the efficacy of results is either ‘positive’ or ‘negative’, with intervention_arm as starting node and outcome as ending node, built based on the columns ‘intervention_group’, ‘outcome_title’, and ‘significance’ from each efficacy result row. ‘Positive’ relationships are shown in green and ‘negative’ relationships are shown in red. Some intervention_arm nodes are connected to a concept node that saves the mapped MeSH term of the intervention_arm title.
- **Outcome**. The outcome node is clinical outcome used in each study. The outcome node can be connected to an outcome_type node to exhibit the types of an outcome, including biomarker, PRO, and clinical endpoint.
- **Adverse_event**. The adverse_event node is reported serious adverse event in each clinical trial. For adverse events, we built the relationship ‘has_ae’ from the column ‘intervention_arm’ to ‘event_title’ from each adverse event result row. Each adverse_event node is connected to an adverse_event_category node, representing the parent biomedical categories of the adverse event name.

We loaded the created nodes and relationships along with their attributes into the graph database Neo4j (database version == 5.3.0).

To summarize, we presented a pipeline to construct a knowledge graph to represent arm-level clinical trial efficacy and safety results. Compared to existing large scale trial databases such as AACT and CTKG, we curated a result database containing identified comparable arm groups in results section. We also integrated efficacy and safety results by matching corresponding arm groups, providing an evidence dataset for evaluating clinical interventions. We constructed a knowledge graph based on the dataset, offering a data infrastructure for further analysis and applications. However, current limitations include: (1) In efficacy results, multiple testings are not adjusted yet. (2) Multiple arms were not included and classified as interventional and comparator. (3) Current dataset only covers clinical trials with both efficacy and safety results.

## Data Records

We deposited this dataset to a publicly available repository on Figshare^25^. The dataset contains clinical trial efficacy and safety results data in CSV, JSON, and PICKLE formats. We also uploaded two sample files for data exploration.

### Data structure

#### Knowledge graph schema

**Fig. 3:**
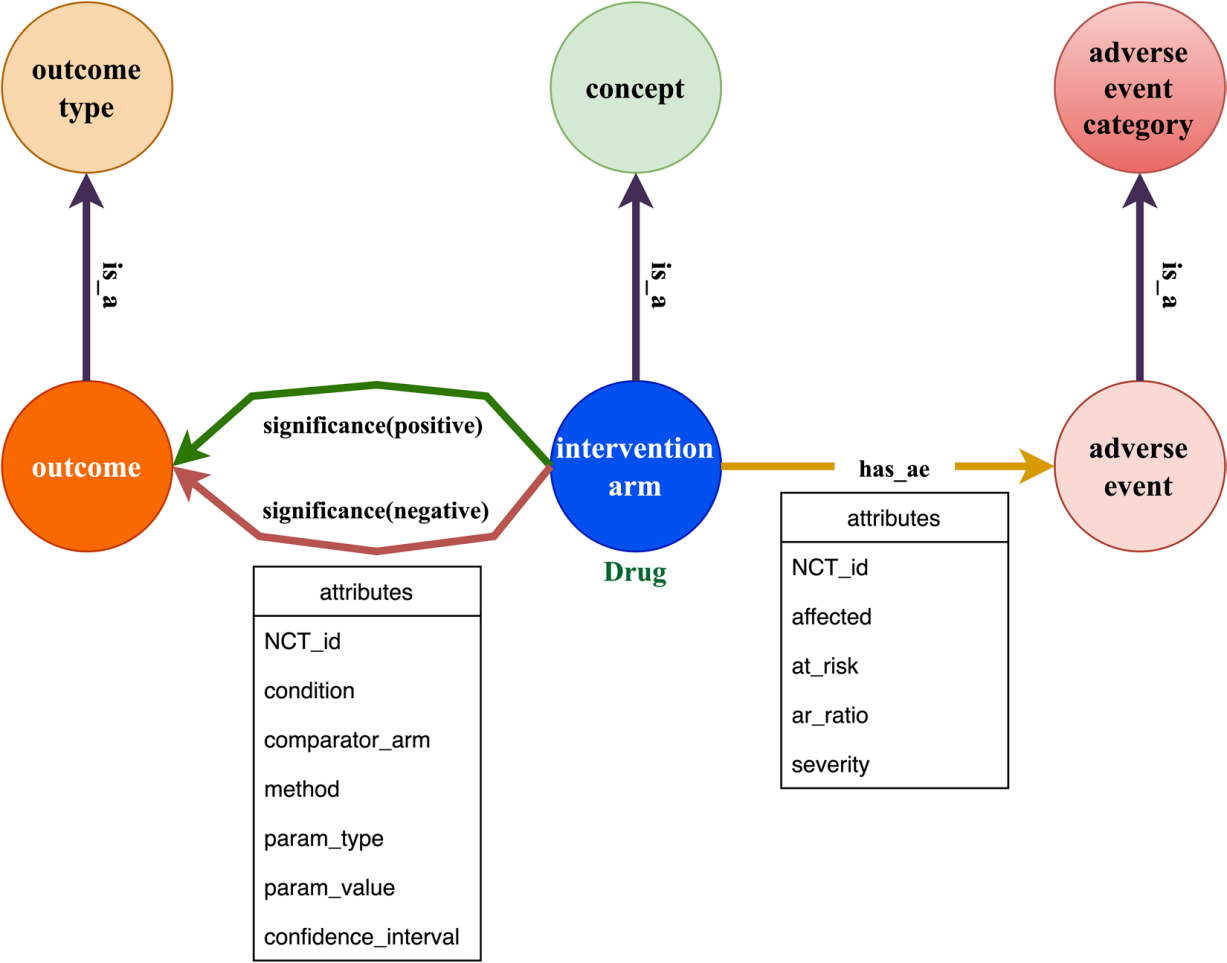
An overview of the schema of arm-level efficacy and safety knowledge graph.

The knowledge graph schema exhibits the most important nodes, relationships, and attributes when evaluating clinical trial results. Compared to the sole ‘has’ relationship in the CTKG, in order to better represent the RESULTS across different studies, we saved all the study-related information of the results in different relationships instead of unique nodes, based on the efficacy and adverse event (Fig. 3). For efficacy, the schema shows the significance, the comparator arm, the parameter value, NCT_id, and condition. For safety, the schema shows the affected/at_risk ratio, NCT_id, and severity. Also, for all the 3 main node types, we applied different techniques described in the method section to give each node type a standardized or taxonomic categories for data integrity and normalization.

### Descriptive statistics

#### Nodes and relationships

Table 4 lists the statistics of nodes and relationships of the knowledge graph based on the above schema. The relation count of efficacy is the number of results with valid intervention group, comparator group and statistical significance. The relation count of safety is the number of serious adverse events with valid arm group and event title. 119,968 comparable efficacy and 803,052 safety relationships are built on reported results from 8,665 studies.

**Table 4:** Statistics of nodes and efficacy/safety relationships.

Node type 0	Count 0	Node type 1	Count 1	Relation name	Relation count
Intervention_arm	10,364	Outcome	41,708	positive/negative	119,968
Intervention_arm	10,364	Adverse_event	40,408	has_ae	803,052
Intervention_arm	10,364	Concept	1,439	Is_a	49,764
Outcome	41,708	Outcome type	3	Is_a	76,575
Adverse event	40,408	Adverse event category	28	Is_a	803,052

#### Outcome classification

25,758 (61.03%) unique outcomes were successfully identified as biomarker, PRO, or clinical endpoint, contained in 76,575 (63.83%) efficacy results. We noticed that there existed differences of the coverage of outcome categories between primary outcomes and secondary outcomes. Table 5 shows the detailed coverage numbers of biomarkers, PROs, and clinical endpoint outcomes.

**Table 5:** Coverage statistics of identified categories in primary and secondary outcomes (‘result’ means numbers of efficacy results, ‘outcome’ means numbers of unique outcomes in efficacy results).

	Primary (result)	Secondary (result)	Primary (outcome)	Secondary (outcome)
Total	23,252	92,966	10,624	30,408
Biomarker	9,321 (40.09%)	29,931 (32.20%)	4,105 (38.64%)	9,702 (31.91%)
PRO	4,378 (18.83%)	27,838 (29.94%)	2,072 (19.50%)	7,501 (24.67%)
Clinical	4,133 (17.77%)	18,700 (20.11%)	2,080 (19.58%)	5,537 (18.21%)
coverage	14,388 (61.88%)	59,738 (64.26%)	6,609 (62.21%)	18,429 (60.61%)

#### Efficacy

**Fig. 4:**
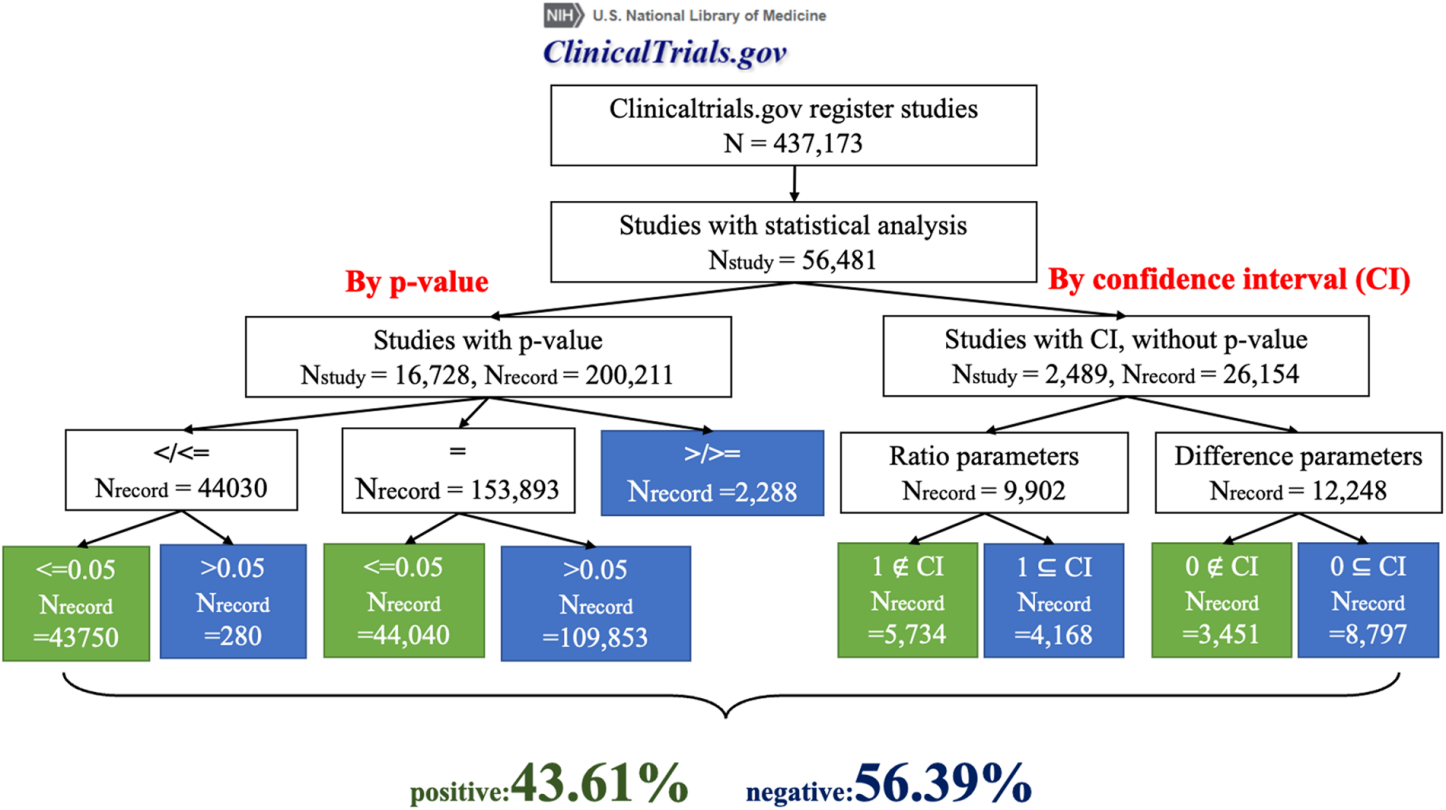
The pipeline and numbers of classification of positive vs. negative results.

To determine the efficacy or statistical significance of an intervention on an outcome, we used p-values and confidence intervals. However, these fields lacked clarity and uniformed formatting. To transform the uncleaned string data into a computable format for our rule-based algorithm, we removed blanks in the strings, transformed the numbers to numeric, and processed the mathematical operators in standard representations. After using the classification pipeline, as we introduced in the method section, we eventually distinguished 56.39% statistically negative results, and 43.61% statistically positive results (Fig. 4).

We also investigated the ratio of positive and negative efficacy results across different dimensions (Table 6). The table reveals varying efficacy of medical interventions across different outcome types, intervention types, and health problems. Among outcome types, choosing biomarker as the outcome is related to the highest 48.33% statistically positive ratio. Compared to other types of intervention such as behavioral and dietary supplement, there exists larger proportions of positive efficacy results related to drug and biological interventions (44.49% and 43.45%, respectively). The efficacy of treatments varies greatly among health problems, with some areas like nutritional and metabolic diseases showing more positive outcomes (53.33%), while others like mental disorders and nervous system diseases have predominantly negative results (65.52% and 70.88%, respectively).

**Table 6:** Distribution of positive and negative efficacy results across different dimensions.

	Positive	Negative
**Outcome type**		
Biomarker	14,166 (48.33%)	15,148 (51.67%)
Patient-reported outcome	12,647 (37.70%)	20,900 (62.30%)
Clinical endpoint	9,820 (41.65%)	13,757 (58.35%)
**Intervention type**		
Drug	43,521 (44.49%)	54,294 (55.51%)
Biological	5,507 (43.45%)	7,168 (56.55%)
Radiation	57 (28.50%)	143 (71.50%)
Behavioral	1,019 (30.02%)	2,375 (69.98%)
Genetic	18 (25.35%)	53 (74.65%)
Other	511 (34.60%)	966 (65.40%)
Device	1,223 (38.92%)	1,919 (61.08%)
Dietary Supplement	155 (28.49%)	389 (71.51%)
Procedure	147 (27.89%)	380 (72.11%)
Combination Product	71 (59.66%)	48 (40.34%)
Diagnostic Test	1 (25.00%)	3 (75.00%)
**Health Problems**		
Infections	2,640 (41.90%)	3,660 (58.10%)
Neoplasms	1,152 (37.39%)	1,929 (62.61%)
Musculoskeletal Diseases	4,338 (45.92%)	5,108 (54.08%)
Digestive System Diseases	1,007 (42.69%)	1,352 (57.31%)
Stomatognathic Diseases	544 (45.18%)	660 (54.82%)
Respiratory Tract Diseases	4,520 (41.63%)	6,337 (58.37%)
Otorhinolaryngologic Diseases	520 (44.67%)	644 (55.33%)
Nervous System Diseases	2,090 (29.12%)	5,086 (70.88%)
Eye Diseases	478 (33.71%)	940 (66.29%)
Urogenital Diseases	1,438 (49.60%)	1,461 (50.40%)
Female Urogenital Diseases and Pregnancy Complications	1,614 (49.89%)	1,621 (50.11%)
Cardiovascular Diseases	2,071 (50.11%)	2,647 (56.10%)
Hemic and Lymphatic Diseases	434 (40.45%)	639 (59.55%)
Congenital, Hereditary, and Neonatal Diseases and Abnormalities	1,398 (51.34%)	1,325 (48.66%)
Skin and Connective Tissue Diseases	3,539 (47.77%)	3,870 (52.23%)
Nutritional and Metabolic Diseases	6,091 (53.33%)	5,331 (46.67%)
Endocrine System Diseases	3,546 (52.64%)	3,190 (47.36%)
Immune System Diseases	4,425 (41.96%)	6,122 (58.04%)
Pathological Conditions, Signs and Symptoms	5,483 (41.23%)	7,817 (58.77%)
Occupational Diseases	8 (4.40%)	174 (95.60%)
Chemically-Induced Disorders	107 (41.63%)	150 (58.37%)
Wounds and Injuries	131 (28.35%)	331 (71.65%)
Mental Disorders	2,408 (34.48%)	4,575 (65.52%)

#### Safety

We investigated the number of affected subjects of SAEs related to the arm groups. In this dataset, after removing events titled ‘Total, serious adverse events’, a total of 2,106,063 subjects (not necessarily unique individual subjects) were affected by SAEs, and 3,538,554 were at risk. Table 7 shows the ranked intervention types of arm groups by the number of affected subjects. Table 7 only included SAEs with valid one-to-one mesh terms and types, meaning records with zero or multiple intervention types were excluded. Note that this is not a causal relationship between SAE and interventions. The reasons of appearances of SAEs can be possibly related to the subjects’ personal health condition, trial design, disease complications, etc. Table 8 lists SAE categories ranked by the number of related affected subjects.

**Table 7:** Numbers of affected subjects by different intervention types.

Intervention_type	Number of affected subjects
Drug	221,278
Biological	24,578
Device	1,060
Behavioral	215
Dietary Supplement	250
Radiation	215
Other	169
Procedure	67
Combination Product	8
Genetic	6

**Table 8:** Numbers of affected subjects by SAE categories.

Category name	Number of affected subjects
Infections and infestations	294,602
Cardiac disorders	286,035
General disorders	195,018
Gastrointestinal disorders	168,440
Respiratory, thoracic and mediastinal disorders	147,184
Neoplasms benign, malignant and unspecified	132,976
Nervous system disorders	129,127
Injury, poisoning and procedural complications	104,784
Vascular disorders	93,766
Blood and lymphatic system disorders	78,164
Metabolism and nutrition disorders	72,235
Musculoskeletal and connective tissue disorders	70,352
Renal and urinary disorders	69,560
Investigations	58,024
Surgical and medical procedures	46,785
Hepatobiliary disorders	32,128
Psychiatric disorders	31,207
Skin and subcutaneous tissue disorders	20,295
Eye disorders	17,144
Reproductive system and breast disorders	16,274
Pregnancy, puerperium and perinatal conditions	10,356
Endocrine disorders	8,578
Immune system disorders	8,405
Ear and labyrinth disorders	5,646
Congenital, familial and genetic disorders	4,496
Social circumstances	2,242
Product Issues	2,240

**Fig. 5:**
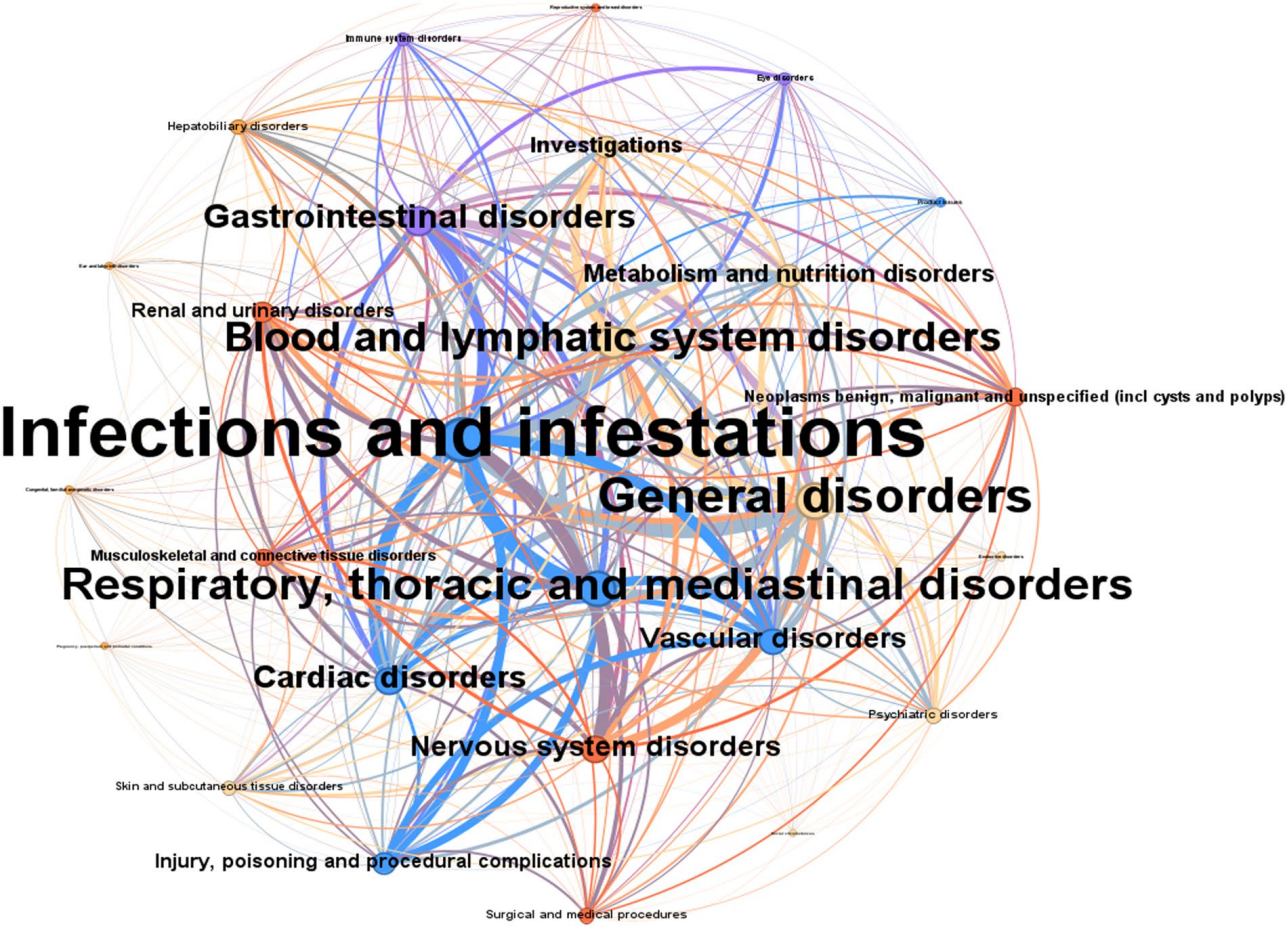
Co-occurrence map of SAE categories.

Also note that this is only the safety results that from studies having efficacy results at the same time, for the purpose of tracking original study for integrating efficacy and safety analysis. We imported the co-occurrence matrix to a network visualization software Gephi^26^ and output the co-occurrence map of SAE categories (Fig. 5). In the co-occurrence map, each node represents an adverse event category. Each edge represents the existence of co-occurrence between two categories. The width of edges represents the computed weighted co-occurrence scores. The size of nodes represents the sum of the scores. The co-occurrence between SAE category ‘Respiratory, thoracic and mediastinal disorders’ and ‘Infections and infestations’ has the highest degree of co-occurrence, meaning it is the most frequently and strongly co-occurred serious adverse event pair in the included clinical trials.

#### Knowledge graph: Antibiotic and infection

**Fig. 6:**
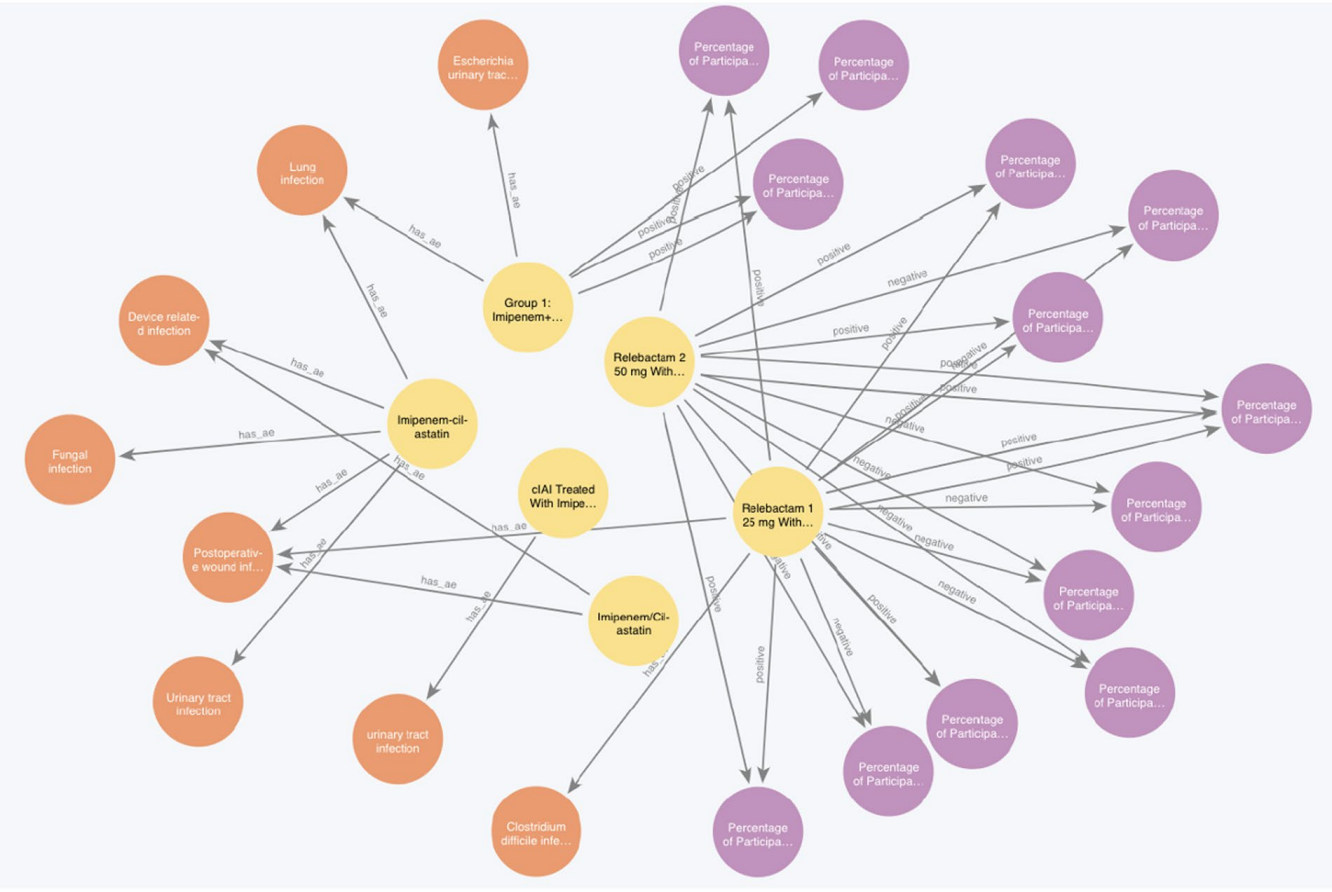
An example KG that shows the case of infection-related adverse events while intervening with the antibiotic imipenem/cilastatin to treat infections. Orange nodes are SAEs, yellow nodes are interventional arm groups, purple nodes are outcomes, visualized in Neo4j Bloom.

In order to present the functionality of evaluating efficacy and safety of an intervention based on the constructed knowledge graph, we retrieved a sub-graph based on a clinical scenario: serious adverse events related to infections while antibiotic ‘imipenem/cilastatin’ being used to treat infections. We queried the knowledge graph, retrieved efficacy results by matching the ‘intervention_group’ and ‘intervention_MeSH’ with ‘imipenem’ or ‘cilastatin’, and retrieved adverse event results by matching the group and adverse events title ‘event_title’ and parent category ‘category’ with the keyword ‘infection’. This dual-faceted inspections of both efficacy and safety are crucial in clinical studies, especially with the population condition and potential adverse events belonging to the same clinical condition. The queried relationships are shown in Fig. 6. The infection-related SAEs give us evidence which types of infections should be paid attention when treated with imipenem/cilastatin.

## Technical Validation

We introduced the full pipeline from data collection, data processing to the final knowledge graph in details. The knowledge graph and original dataset are transparent in the following aspects: 1) The origin of data comes from CT.gov, which is an open-access platform that everyone is able to download the full structured dataset. 2) The process of formation of the knowledge graph is reproducible. The codes will be available to access publicly. 3) Users are able to adjust the knowledge graph based on provided codes and sub-datasets, providing the flexibility to alter the data based on their own research interests.

Firstly, we acknowledge that conducting validation in natural language processing (NLP) is inherently challenging, primarily due to the lack of gold standard corpus. Constructing such a corpus for NLP algorithm validation is typically a time-consuming and labor-intensive task. Nevertheless, we made three attempts through a systematic literature search. We have identified two independent works done by other research groups related to CT.gov reported results analysis, which serve as our reference of alignment.

### Reliability validation

Next, we present three approaches to validate the **accuracy** for efficacy determination, **consistency** with external resources, and **feasibility** for potential safety data resource.

#### Manual random sample checkup

We validated the applied BioBert algorithm with a manually-annotated dataset by separating the groups manually. To build this validation dataset, we randomly selected the un-separated groups from 100 efficacy result records, and manually inspected the comparison information from the study design on CT.gov. Manually separated ground truth intervention group and comparator group are stored as columns ‘inter_label’ and ‘comp_label’. To evaluate the algorithm, we check whether the BioBert-extracted ‘intervention_group’ matches the ground truth ‘inter_label’, also for ‘comparator_group’ and ‘comp_label’. The accuracy (correctly mapped) for the intervention group and comparator group is 84% and 80%, respectively. This manually-annotated dataset has been deposited to the Figshare repository.

#### Validation with a large-scale P-values research

To investigate manipulation of p-values in clinical trials, recent research collected 12,621 P-values in primary outcomes of 4,977 clinical trials from 2007 to 2019 in AACT^27^. From our dataset, we gathered 11,216 efficacy results with valid P-values in 4,222 clinical trials after filtering by the same condition (2007 to 2019, phase 2/3, primary outcome, valid P-value), showing in consistency with 88.9% of the results and 84.83% of the number of trials from the existing study. Note that all of our efficacy results are in a comparable format, enabling conditional efficacy analysis and fast evidence synthesis.

#### Validation with safety analysis

**Fig. 7:**
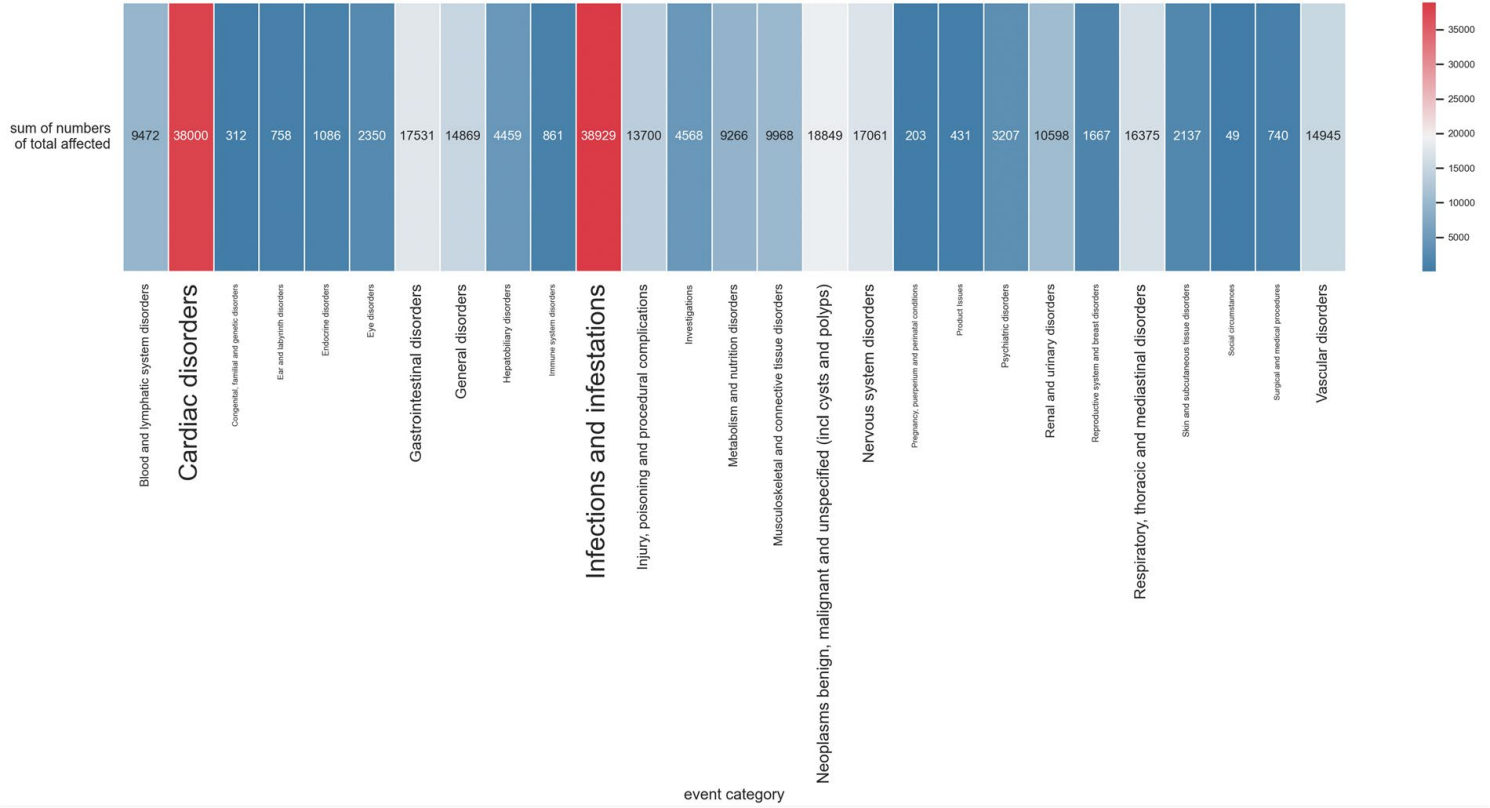
The distribution of health problems of serious adverse events by the number of affected subjects.

To systematically validate the feasibility for an investigation of safety results in clinical trials, we went through three related representative studies that conducted quantitative analysis of safety results or serious adverse events^3,28,29^. Unfortunately, all the studies are manually collected with small sample size. Also, the related datasets are not publicly available. There also exists an inconsistency between safety results in published research, Drugs@FDA, and CT.gov^3,28,29^. Thus, we could not perform a systematic comparison of our work with the other resources. Nevertheless, we compared the distribution of affected subjects by SAEs in CT.gov with an analysis of reported adverse events in FAERS (FDA’s Adverse Event Reporting System) from a data visualization community (https://public.tableau.com/app/profile/simon.lafosse/viz/WYMD-Top20v0_5/WYMD). We reproduced the distribution from 2018 to 2019 and visualized by SAE category (Fig. 7). The units used are different (number of affected subjects versus number of adverse events records). Nonetheless, it validates the feasibility for a potential large-scale safety results dataset.

### Validation of the reproducibility of the dataset and method

In this study, we adopted an automatic and transparent pipeline applying commonly used tools such as MTI^19,20^. The highly automated process of our method guarantees the reproducibility of the dataset and method. The primary data source is also an open-sourced dataset with high availability provided by NLM in XML format.

In summary, we curated a dataset beyond the meta-data of CT.gov^30^. This was achieved by linking efficacy with safety results at the experimental arm group level within each trial, and connecting them across all trials through a knowledge graph. Following the computable principle of “calculate once, use many times” we anticipate that this structured dataset will enhance the reuse and interpretation of CT.gov results data. Regular updates to the dataset, occurring every two months, are planned. Our approach effectively connects the generally described searchable information and the specifically detailed yet under-utilized reported results.

Through technical validation aligned with other relevant studies, we affirm that CT.gov can serve as a potential valuable data source for monitoring adverse drug events. This resource is expected to facilitate a more systematic and feasible practice of evidence synthesis and health technology assessment, by incorporating both positive and negative results, distinguishing biomarkers, patient-reported outcomes, with clinical endpoints, while also maintaining a balanced consideration of both efficacy and safety outcomes for a given medical intervention.

### Supplementary information

Table S1

## Data Availability

The source codes of data collection, processing and analysis are stored at: (https://github.com/xuanyshi/Finer-Grained-Clinical-Trial-Results).
