# Supplementary material for: Constructing a finer-grained representation of clinical trial results from ClinicalTrials.gov
Source: Sci Data. 2024 Jan 6;11:41. doi: 10.1038/s41597-023-02869-7 (PMC10771511; doi:10.1038/s41597-023-02869-7)
Supplement: Supplementary file 1 — Table S1 [file 41597_2023_2869_MOESM1_ESM.docx]

Table S1. Positive and negative biomarker outcome set of each health problem

| **health_problem**  **_mesh** | **health_problem_title** | **outcome_title** | **positive_num** | **outcome_title** | **negative_num** |
| --- | --- | --- | --- | --- | --- |
| C01 | Infections | Geometric Mean Titer of Serotype-specific Opsonophagocytic Activity (OPA) Following Vaccination With Separate V114 Lots | 45 | Percentage of Participant With Normal Alanine Aminotransferase (ALT) Levels | 40 |
| C01 | Infections | Percentage of Participants Achieving the Serotype-specific Pneumococcal Immunoglobulin G (IgG) Antibody Threshold Value of ≥0.35 µg/mL for the 13 Common Serotypes in V114 and Prevnar 13™: 1 Month Post Vaccination 3 | 26 | Percentage of Participants Who Were HBeAg Negative | 33 |
| C01 | Infections | Ratio to Baseline in Renal Biomarkers-Urine and Serum Beta-2 Microglobulin (B2M), Urine Albumin/Creatinine, Urine B2M/Urine Creatinine, Urine Phosphate, Urine Protein/Creatinine, Urine RBP 4 and Urine RBP 4/Urine Creatinine at Weeks 24, 48 | 22 | Change From Baseline in HBsAg Levels at Weeks 4, 8, 16, 28, 40, 52, 64, 76, 88, and 100 | 31 |
| C01 | Infections | Serotype-specific Opsonophagocytic Activity (OPA) Geometric Mean Titers (GMTs) 1 Month After Vaccination for 12 Common Serotypes in 13vPnC/23vPS Group Relative to 23vPS Group and 23vPS/23vPS Group | 21 | Serotype-specific Geometric Mean Concentration of IgG Antibody | 21 |
| C01 | Infections | Comparison of Serotype-specific Geometric Mean Concentration of IgG Antibody Response 4 Weeks After a 3-dose Primary Series to 4 Weeks After a Booster Dose | 20 | Change From Baseline in Hepatitis B Virus Deoxyribonucleic Acid (HBV DNA) Levels at Weeks 4, 8, 16, 28, 40, 52, 64, 76, 88, and 100 | 19 |
| C04 | Neoplasms | Progression-Free Survival (PFS) | 40 | PFS in Subgroups That Were Defined by Germline PDGFRB Polymorphisms | 48 |
| C04 | Neoplasms | AUC(0-2h) of [6R]-5,10-methylene-THF | 6 | OS in Subgroups That Were Defined by Germline PDGFRB Polymorphisms | 46 |
| C04 | Neoplasms | AUC(0-2h) of [6S]-5-THF | 6 | PFS in Subgroups That Were Defined by RNA Expression Profile | 35 |
| C04 | Neoplasms | AUC(0-2h) of [6SR]-5-formyl-THF | 6 | Overall Survival (OS) in Subgroups That Were Defined by Germline VEGFR2 Polymorphisms | 24 |
| C04 | Neoplasms | AUC(Last) of [6R]-5,10-methylene-THF | 6 | PFS in Subgroups That Were Defined by Germline VEGFR2 Polymorphisms | 22 |
| C05 | Musculoskeletal Diseases | Change From Baseline in Serum Uric Acid Levels at Day 1, Day 3, Day 7, Day 11, Day 14 and Follow-up | 44 | Change From Baseline in Plasma Levels of Hypoxanthine at Day 1, Day 7, Day 14, and at Follow-up | 36 |
| C05 | Musculoskeletal Diseases | Change From Baseline in Erythrocyte Sedimentation Rate (ESR) at Weeks 2, 4, 8, 12, 16, 20, 24, 32, 40, 48, and 52 | 27 | Current Pain During the Hospital Stay Assessed by the Pain NRS | 26 |
| C05 | Musculoskeletal Diseases | Change From Baseline in C-reactive Protein (CRP) at Weeks 2, 4, 8, 12, 16, 20, 24, 32, 40, 48, and 52 | 24 | Change From Baseline in Predictive Biomarkers: Amyloid A, Chemokine (C-C Motif) Ligand 17, Chemokine (C-X-C Motif) Ligand 13, Interleukin 6, Macrophage-Derived Chemokine | 26 |
| C05 | Musculoskeletal Diseases | Change From Baseline in Plasma Levels of Xanthine at Day 1, Day 7, Day 14, and at Follow-up | 22 | Change From Baseline in Plasma Levels of Xanthine at Day 1, Day 7, Day 14, and at Follow-up | 20 |
| C05 | Musculoskeletal Diseases | Percent Change From Baseline in Serum Urate Levels at Week 28. | 13 | Change From Baseline in C-Reactive Protein (CRP) at Weeks 1, 2, 4, 8, 12 and 16 | 18 |
| C06 | Digestive System Diseases | Change From Baseline in Quantitative Hepatitis B Surface Antigen (Log qHBsAg) Over Time | 17 | Percentage of Participant With Normal Alanine Aminotransferase (ALT) Levels | 40 |
| C06 | Digestive System Diseases | Change From Baseline in Hepatitis B Virus Deoxyribonucleic Acid (HBV DNA) Levels at Weeks 4, 8, 16, 28, 40, 52, 64, 76, 88, and 100 | 14 | Percentage of Participants Who Were HBeAg Negative | 33 |
| C06 | Digestive System Diseases | Absolute Change in Percent Predicted Forced Expiratory Volume in 1 Second (ppFEV1) | 10 | Change From Baseline in HBsAg Levels at Weeks 4, 8, 16, 28, 40, 52, 64, 76, 88, and 100 | 31 |
| C06 | Digestive System Diseases | Progression Free Survival (PFS) | 6 | Change From Baseline in Hepatitis B Virus Deoxyribonucleic Acid (HBV DNA) Levels at Weeks 4, 8, 16, 28, 40, 52, 64, 76, 88, and 100 | 19 |
| C06 | Digestive System Diseases | AUC(0-2h) of [6R]-5,10-methylene-THF | 6 | Progression Free Survival (PFS) | 6 |
| C07 | Stomatognathic Diseases | AUCgly(0-90) for Test Zinc-IPMP, Test Zinc Non-IPMP, Positive Control, Non-SLS Negative Control and SLS Negative Control | 6 | Anti-S.Pneumoniae Antibody Concentration in US Sub-cohort of Pooled MMR Groups | 12 |
| C07 | Stomatognathic Diseases | Enamel Fluoride Uptake | 5 | AUClive:Dead(0-90) for Test Zinc-IPMP, Test Zinc Non-IPMP, Positive Control, Non-SLS Negative Control and SLS Negative Control | 10 |
| C07 | Stomatognathic Diseases | AUCregrowth(0-90) for Test Zinc-IPMP, Test Zinc Non-IPMP, Positive Control, Non-SLS Negative Control and SLS Negative Control | 4 | AUCregrowth(0-90) for Test Zinc-IPMP, Test Zinc Non-IPMP, Positive Control, Non-SLS Negative Control and SLS Negative Control | 6 |
| C07 | Stomatognathic Diseases | Adjusted Mean Percent Net Erosion Resistance (NER) of Enamel Specimens Exposed to Test Dentifrice + Test MR Relative to: 1) Test Dentifrice+ Sterile Water Rinse 2) Reference Dentifrice+ Sterile Water Rinse 3) Placebo Dentifrice+ Sterile Water Rinse | 3 | AUCgly(0-90) for Test Zinc-IPMP, Test Zinc Non-IPMP, Positive Control, Non-SLS Negative Control and SLS Negative Control | 3 |
| C07 | Stomatognathic Diseases | Adjusted Mean Percentage Surface Microhardness (SMH) Recovery of Enamel Specimens Exposed to Test Dentifrice + Test MR Relative to: 1)Test Dentifrice+Sterile Water Rinse 2)Reference Dentifrice+Sterile Water Rinse 3)Placebo Dentifrice+ Sterile Water Rinse | 3 | Number of Participants With Unplanned Breaks in Cisplatin Chemotherapy Treatment | 2 |
| C08 | Respiratory Tract Diseases | Geometric Mean Titer of Serotype-specific Opsonophagocytic Activity (OPA) Following Vaccination With Separate V114 Lots | 45 | Change From Baseline in Specific Imaging Airway Volume (siVaw), Measured at FRC and TLC Scan Conditions, Presented in Longitudinal and Scan Trimmed Scan Types, Measured in 5 Lobes and 5 Regions at Screening, Day 12 and Day 28 | 100 |
| C08 | Respiratory Tract Diseases | Change From Baseline in Fraction of Exhaled Nitric Oxide (FeNO) Over Time Following the Cessation of Repeat Dose Treatment With FF/VI | 34 | Change From Baseline in Imaging Airways Volume: iVaw, Measured at FRC and TLC Scan Conditions, Presented in Longitudinal and Scan Trimmed Scan Types, Measured in 5 Lobes and 5 Regions at Screening, Day 12 and Day 28 | 100 |
| C08 | Respiratory Tract Diseases | Change From Baseline in Morning and Evening Peak Expiratory Flow Rate (PEF) Over 26 and 52 Weeks of Treatment | 28 | Change From Baseline in Imaging Airways Resistance ( iRaw) Measured at FRC and TLC Scan Conditions, Presented in Scan Trimmed Scan Types, Measured in 5 Lobes and 5 Regions at Screening, Day 12 and Day 28 | 60 |
| C08 | Respiratory Tract Diseases | Comparison of Serotype-specific Geometric Mean Concentration of IgG Antibody Response 4 Weeks After a 3-dose Primary Series to 4 Weeks After a Booster Dose | 20 | Change From Baseline in Imaging Specific Airways Resistance: siRaw Measured at FRC and TLC Scan Conditions, Presented in Scan Trimmed Scan Types, Measured in 5 Lobes and 5 Regions at Screening, Day 12 and Day 28 | 60 |
| C08 | Respiratory Tract Diseases | Comparison of Serotype-specific Geometric Mean Concentration of IgG Antibody Response 4 Weeks After a Booster Dose to One Year After a Booster Dose | 20 | PFS in Subgroups That Were Defined by Germline PDGFRB Polymorphisms | 48 |
| C09 | Otorhinolaryngologic Diseases | Change From Baseline in HDM-specific IgE Levels at Week 8 | 4 | Change From Baseline in AM and PM Peak Expiratory Flow Rate (PEFR) | 11 |
| C09 | Otorhinolaryngologic Diseases | Pain Intensity (PI) as Rated on a 6-point VRS by the Patient at 0.5, 1, 2 and 3 Hours After the First Lozenge | 3 | Change From Baseline in Forced Expiratory Volume in One Second (FEV1) | 3 |
| C09 | Otorhinolaryngologic Diseases | the Effect of Squeezable Bottle and Syringe on Clinical Effectiveness in Sinusitis Children | 2 | Change From Baseline in Forced Vital Capacity (FVC) | 3 |
| C09 | Otorhinolaryngologic Diseases | Percentage of Participants Who Discontinued Study Drug Due to an Adverse Event | 2 | Change From Baseline in Forced Expiratory Flow (FEF) Between 25% and 75% of the Vital Capacity (FEF25%-75%) | 3 |
| C09 | Otorhinolaryngologic Diseases | Total Nasal Airflow on Day 8 Measured Using Active Anterior Rhinomanometry (AAR) | 2 | Change From Baseline 24-hour Urinary Free Cortisol Level | 3 |
| C10 | Nervous System Diseases | CSF IL-6, sTREM2, HMGB1, Albumin, IgG | 10 | Geometric Mean Titers (GMTs) of Meningococcal Serogroups A, C, Y, And W Antibodies Following Vaccination With 3 Lots of MenACYW Conjugate Vaccine | 12 |
| C10 | Nervous System Diseases | ITP: Number of Combined Unique Active (CUA) Lesions, New or Enlarging Time Constant 2 (T2) Lesions, and New or Persisting Time Constant 1 (T1) Gadolinium Enhanced (Gd+) Lesions Per Participant Per Scan | 6 | Percentage of Participants Achieving hSBA Vaccine Seroresponse for Meningococcal Serogroups A, C, Y And W Following Vaccination With 3 Lots of MenACYW Conjugate Vaccine | 10 |
| C10 | Nervous System Diseases | Mean Blood Flow Velocity in Middle Cerebral Artery | 5 | Incidence of Non-delirium Complications After Surgery | 8 |
| C10 | Nervous System Diseases | Total Cumulative Prednisone Dose | 4 | Change From Baseline in Peak Work (in Watts/kg) During Exercise Testing at Week 12 in Part 1 | 7 |
| C10 | Nervous System Diseases | Statistically Significant Change From Baseline to 12 Week Endpoint in Laboratory Measures - Chloride, High Density Lipoprotein, Sodium, and Triglycerides | 4 | Change From Baseline in Respiratory Function Tests to Characterize the Degree of Involvement of Respiratory Muscles. | 4 |
| C11 | Eye Diseases | Distribution of Change in Visual Acuity (Letters) From Baseline to 1 Year | 8 | Percentage of Ranibizumab Re-injections Received Over 28 and 52 Weeks | 10 |
| C11 | Eye Diseases | Ex Vivo Total Cholesterol Uptake at Day 30 | 8 | Change From Baseline in GA Area in Complement Factor I (CFI) Positive and Negative Participants at Week 48 | 7 |
| C11 | Eye Diseases | Mean Number of Macular Laser Treatments From Baseline Through Months 24 and 36 | 4 | Percentage of Participants Avoiding a Loss of ≥15, ≥10, or ≥5 Letters From the Baseline BCVA in the Study Eye Averaged Over Weeks 40, 44, and 48 | 6 |
| C11 | Eye Diseases | Change From Baseline in Visual Acuity (VA): Double Masked Phase | 4 | Distribution of Change in Visual Acuity (Letters) From Baseline to 1 Year | 4 |
| C11 | Eye Diseases | Change From Baseline in Central Retinal Thickness (CRT) at Week 52 as Assessed on Optical Coherence Tomography (OCT) - LOCF | 4 | Loss of Visual Acuity | 3 |
| C12 | Urogenital Diseases | Secondary Efficacy Endpoints - Vaginal Mucosa Assessment (Vaginal Color) | 12 | Systolic, Diastolic and Mean Blood Pressure at Week 0, 3, 6, 12, and 16 | 12 |
| C12 | Urogenital Diseases | Change From Baseline to Day 90 in Serum Potassium | 7 | Blastocyst Quality, Intention-to-treat (ITT) Analysis Set | 7 |
| C12 | Urogenital Diseases | Change From Baseline in eGFR | 7 | Creatinine Clearance (CrCl) in the Immediate Post-transplant Period | 6 |
| C12 | Urogenital Diseases | Progression-Free Survival (PFS) | 5 | Pharmacokinetics: AUC(0-∞) of Tadalafil | 6 |
| C12 | Urogenital Diseases | Percent Change From Baseline to 12 Week Endpoint in High-Density Lipoprotein Cholesterol (HDL-C) and Low-Density Lipoprotein Cholesterol (LDL-C) | 4 | Change From Baseline to 12 Week Endpoint in Total Cholesterol and Triglycerides | 5 |
| C13 | Female Urogenital Diseases and Pregnancy Complications | Secondary Efficacy Endpoints - Vaginal Mucosa Assessment (Vaginal Color) | 12 | Systolic, Diastolic and Mean Blood Pressure at Week 0, 3, 6, 12, and 16 | 12 |
| C13 | Female Urogenital Diseases and Pregnancy Complications | Change From Baseline to Day 90 in Serum Potassium | 7 | Blastocyst Quality, Intention-to-treat (ITT) Analysis Set | 7 |
| C13 | Female Urogenital Diseases and Pregnancy Complications | Change From Baseline in eGFR | 7 | Creatinine Clearance (CrCl) in the Immediate Post-transplant Period | 6 |
| C13 | Female Urogenital Diseases and Pregnancy Complications | Change From AURORA 1 Baseline (i.e., Month 0) in Urine Protein to Creatinine Ratio (UPCR) | 4 | Progression Free Survival (PFS) | 5 |
| C13 | Female Urogenital Diseases and Pregnancy Complications | Change From AURORA 1 Baseline (i.e., Month 0) in Urine Protein | 4 | Change From Baseline to Day 90 in eGFR | 5 |
| C14 | Cardiovascular Diseases | Change From Baseline in Daytime (6am to 10 pm) Mean Systolic Blood Pressure Measured by Ambulatory Blood Pressure Monitoring. | 27 | Inflammatory Mediator Levels, Interleukin-1b, Interleukin 6 and Tumor Necrosis Factor (TNF) (pg/ml) | 14 |
| C14 | Cardiovascular Diseases | Change From Baseline in Daytime (6am to 10 pm) Mean Diastolic Blood Pressure Measured by Ambulatory Blood Pressure Monitoring. | 24 | Change From Baseline in Mean Ambulatory Blood Pressure During the Final 2, 4, and 6 Hours of the Dosing Interval at Week 4 | 12 |
| C14 | Cardiovascular Diseases | Change From Baseline in the Trough (22-24-hr) Mean Diastolic Blood Pressure Measured by Ambulatory Blood Pressure Monitoring. | 23 | Change From Baseline in Plasma Concentration of Serum N-terminal Pro-Brain Natriuretic Peptide (NT-proBNP) at Months 5, 9, 13, 17, 21, 25, 29, 33, 37, 42, 48 and Final Visit | 12 |
| C14 | Cardiovascular Diseases | Change From Baseline in the Trough (22-24-hr) Mean Systolic Blood Pressure Measured by Ambulatory Blood Pressure Monitoring. | 22 | Change From Baseline in Urine Albumin-to-Creatinine Ratio at Months 5, 9, 13, 17, 21, 25, 29, 33, 37, 42, 48 and Final Visit | 12 |
| C14 | Cardiovascular Diseases | Change From Baseline in the Nighttime (12 am to 6 am) Mean Systolic Blood Pressure Measured by Ambulatory Blood Pressure Monitoring. | 22 | Change From Baseline in the Nighttime (12 am to 6 am) Mean Diastolic Blood Pressure Measured by Ambulatory Blood Pressure Monitoring. | 11 |
| C15 | Hemic and Lymphatic Diseases | Mean Change in Ferritin and Hepcidin From Baseline to the End of the Primary Efficacy Period | 10 | Mean Change in Iron and Total Iron Binding Capacity (TIBC) From Baseline to the End of the Primary Efficacy Period | 7 |
| C15 | Hemic and Lymphatic Diseases | Change From Baseline in Hematocrit | 8 | Mean Change in Hematocrit and Reticulocytes From Baseline to the End of the Primary Efficacy Period | 6 |
| C15 | Hemic and Lymphatic Diseases | Summary of Subgroup Analyses for Kaplan-Meier Estimates for Time to Death From Any Cause | 7 | Change From Baseline in Reticulocyte Count | 6 |
| C15 | Hemic and Lymphatic Diseases | Mean Change in Red Blood Cell (RBC) Count and Absolute Reticulocyte Count From Baseline to the End of the Primary Efficacy Period | 7 | Progression-free Survival (PFS) | 5 |
| C15 | Hemic and Lymphatic Diseases | Mean Change in Hemoglobin (Hb) Levels From Pre-treatment to the End of the Primary Efficacy Period | 6 | Summary of Subgroup Analyses for Kaplan-Meier Estimates for Time to Death From Any Cause | 5 |
| C16 | Congenital, Hereditary, and Neonatal Diseases and Abnormalities | Change From Baseline in Serum Uric Acid Levels at Day 1, Day 3, Day 7, Day 11, Day 14 and Follow-up | 44 | Change From Baseline in Plasma Levels of Hypoxanthine at Day 1, Day 7, Day 14, and at Follow-up | 36 |
| C16 | Congenital, Hereditary, and Neonatal Diseases and Abnormalities | Change From Baseline in Plasma Levels of Xanthine at Day 1, Day 7, Day 14, and at Follow-up | 22 | Change From Baseline in Plasma Levels of Xanthine at Day 1, Day 7, Day 14, and at Follow-up | 20 |
| C16 | Congenital, Hereditary, and Neonatal Diseases and Abnormalities | Percent Change From Baseline in Serum Urate Levels at Week 28. | 13 | Fraction of Inspired Oxygen (FiO2) (Percent) During the First 24 h and up to Day 7 | 11 |
| C16 | Congenital, Hereditary, and Neonatal Diseases and Abnormalities | Percent Change From Baseline in Serum Urate Levels at Final Visit | 13 | Oxygen Requirement and Ventilatory Support -- SpO2/FiO2 Ratio | 10 |
| C16 | Congenital, Hereditary, and Neonatal Diseases and Abnormalities | Absolute Change in Percent Predicted Forced Expiratory Volume in 1 Second (ppFEV1) | 10 | Change From Baseline in Peak Work (in Watts/kg) During Exercise Testing at Week 12 in Part 1 | 7 |
| C17 | Skin and Connective Tissue Diseases | Change From Baseline in Erythrocyte Sedimentation Rate (ESR) at Weeks 2, 4, 8, 12, 16, 20, 24, 32, 40, 48, and 52 | 27 | Change From Baseline in Predictive Biomarkers: Amyloid A, Chemokine (C-C Motif) Ligand 17, Chemokine (C-X-C Motif) Ligand 13, Interleukin 6, Macrophage-Derived Chemokine | 26 |
| C17 | Skin and Connective Tissue Diseases | Change From Baseline in C-reactive Protein (CRP) at Weeks 2, 4, 8, 12, 16, 20, 24, 32, 40, 48, and 52 | 24 | Change From Baseline in C-Reactive Protein (CRP) at Weeks 1, 2, 4, 8, 12 and 16 | 18 |
| C17 | Skin and Connective Tissue Diseases | Change From Baseline in Percentage Body Surface Area at Week 2, 4, 8 and 12 | 8 | Change From Baseline in Flow Cytometry: 6 Colour TB Natural Killer (NK) Panel- CD16+CD56+, CD19, CD3, CD3+CD4+ | 16 |
| C17 | Skin and Connective Tissue Diseases | Percentage of Participants Achieving Investigator's Global Assessment (IGA) Response of Clear (0) or Almost Clear (1) and a Reduction of Greater Than or Equal to (>=) 2 Points From Baseline at Weeks 12, 16, 28, 40, and 52: Double-blind Period | 8 | Change From Baseline in Predictive Biomarkers: Chitinase 3 Like 1, Matrix Metalloproteinase 3 (MMP-3) | 14 |
| C17 | Skin and Connective Tissue Diseases | Percentage of Participants Achieving Patient Global Assessment (PtGA) Response of 'Clear (0)' or 'Almost Clear (1)' and Greater Than or Equal to 2 Points Improvement From Baseline at Weeks 12, 16, 28, 40 and 52: Double-blind Period | 8 | Change From Baseline in Flow Cytometry: CD16+ Monocyte Panel: CD14-HLA-DR+CD11cbr+CD123-, CD14br+CD16+, CD14br+CD16-, CD14lo+CD16br+ | 14 |
| C18 | Nutritional and Metabolic Diseases | Change From Baseline in Serum Uric Acid Levels at Day 1, Day 3, Day 7, Day 11, Day 14 and Follow-up | 44 | 7-Point Self-Monitored Blood Glucose (SMBG) Profiles | 53 |
| C18 | Nutritional and Metabolic Diseases | Change From Baseline in Fasting Plasma Glucose (FPG) | 34 | Change From Baseline in Fasting Lipid Profile (Triglycerides/Cholesterol) | 39 |
| C18 | Nutritional and Metabolic Diseases | Change From Baseline in Carbon Monoxide Diffusion Capacity (DLco) | 27 | Change From Baseline in Plasma Levels of Hypoxanthine at Day 1, Day 7, Day 14, and at Follow-up | 36 |
| C18 | Nutritional and Metabolic Diseases | Change From Baseline in Fasting Plasma Glucose | 22 | Change From Baseline in Carbon Monoxide Diffusion Capacity (DLco) | 26 |
| C18 | Nutritional and Metabolic Diseases | Change From Baseline in Plasma Levels of Xanthine at Day 1, Day 7, Day 14, and at Follow-up | 22 | ADDENDUM: Insulin Dose | 22 |
| C19 | Endocrine System Diseases | Change From Baseline in Fasting Plasma Glucose (FPG) | 34 | 7-Point Self-Monitored Blood Glucose (SMBG) Profiles | 53 |
| C19 | Endocrine System Diseases | Change From Baseline in Carbon Monoxide Diffusion Capacity (DLco) | 27 | Change From Baseline in Carbon Monoxide Diffusion Capacity (DLco) | 26 |
| C19 | Endocrine System Diseases | Change From Baseline in Fasting Plasma Glucose | 22 | ADDENDUM: Insulin Dose | 22 |
| C19 | Endocrine System Diseases | Percent Change From Baseline in ANGPTL3, TC, LDL-C, HDL-C, VLDL-C, Non-HDL-C, ApoB (ApoB-48, ApoB-100), ApoCIII, ApoAI, FFA, and Lp(a) at Primary Analysis Time Point | 20 | Change From Baseline in Fasting Plasma Glucose | 21 |
| C19 | Endocrine System Diseases | Change From Baseline in Forced Expiratory Volume in One Second (FEV1) | 19 | INITIATION: 7-point Self-monitored Plasma Glucose (SMPG) Profiles and Postprandial Excursions | 20 |
| C20 | Immune System Diseases | Change From Baseline in Fraction of Exhaled Nitric Oxide (FeNO) Over Time Following the Cessation of Repeat Dose Treatment With FF/VI | 34 | Change From Baseline in Peak Expiratory Flow (PEF) During Treatment and Following Cessation of Repeat Dose Treatment With FF/VI | 30 |
| C20 | Immune System Diseases | Change From Baseline in Morning and Evening Peak Expiratory Flow Rate (PEF) Over 26 and 52 Weeks of Treatment | 28 | Change From Baseline in Predictive Biomarkers: Amyloid A, Chemokine (C-C Motif) Ligand 17, Chemokine (C-X-C Motif) Ligand 13, Interleukin 6, Macrophage-Derived Chemokine | 26 |
| C20 | Immune System Diseases | Change From Baseline in Erythrocyte Sedimentation Rate (ESR) at Weeks 2, 4, 8, 12, 16, 20, 24, 32, 40, 48, and 52 | 27 | Change From Baseline in C-Reactive Protein (CRP) at Weeks 1, 2, 4, 8, 12 and 16 | 18 |
| C20 | Immune System Diseases | Change From Baseline in C-reactive Protein (CRP) at Weeks 2, 4, 8, 12, 16, 20, 24, 32, 40, 48, and 52 | 24 | 7-Point Self-Monitored Blood Glucose (SMBG) Profiles | 18 |
| C20 | Immune System Diseases | Ratio to Baseline in Renal Biomarkers-Urine and Serum Beta-2 Microglobulin (B2M), Urine Albumin/Creatinine, Urine B2M/Urine Creatinine, Urine Phosphate, Urine Protein/Creatinine, Urine RBP 4 and Urine RBP 4/Urine Creatinine at Weeks 24, 48 | 22 | Change From Baseline in Flow Cytometry: 6 Colour TB Natural Killer (NK) Panel- CD16+CD56+, CD19, CD3, CD3+CD4+ | 16 |
| C23 | Pathological Conditions, Signs and Symptoms | Cumulative Percentage of Participants With Treatment Failure | 72 | Cumulative Percentage of Participants With Treatment Failure | 46 |
| C23 | Pathological Conditions, Signs and Symptoms | Pain Intensity Difference (PID) | 47 | Change From Baseline in Fasting Lipid Profile (Triglycerides/Cholesterol) | 39 |
| C23 | Pathological Conditions, Signs and Symptoms | Time-weighted Sum of Pain Intensity Difference (SPID) | 18 | Pain Intensity Difference (PID) | 20 |
| C23 | Pathological Conditions, Signs and Symptoms | Percentage of Participants With Treatment Failure | 15 | Non-opioid Rescue Medication - Ibuprofen | 16 |
| C23 | Pathological Conditions, Signs and Symptoms | Time to Treatment Failure | 12 | Non-opioid Rescue Medication - Paracetamol | 15 |
| C24 | Occupational Diseases | Total Sleep Time Measured by Actigraphy (Nights 6-7) | 1 | Latency to Persistent Sleep Over a 2-night Average Measured by Polysomnography (Nights 6-7) | 3 |
| C24 | Occupational Diseases | Sleep Efficiency Measured by Actigraphy (Nights 6-7) | 1 | Latency to Persistent Sleep Over a 2-night Average Measured by Polysomnography (Nights 13-14) | 3 |
| C24 | Occupational Diseases | Sleep Latency Measured by Actigraphy (Nights 6-7) | 1 | Total Sleep Time Over a 2-night Average Measured by Polysomnography (Nights 6-7) | 3 |
| C24 | Occupational Diseases | Sleep Time Measured by Actigraphy (Nights 6-7) | 1 | Total Sleep Time Over a 2-night Average Measured by Polysomnography (Nights 13-14) | 3 |
| C24 | Occupational Diseases | Sleep Time Measured by Actigraphy (Nights 13-14) | 1 | Sleep Efficiency Over a 2-night Average Measured by Polysomnography (0-3 Hours) (Nights 6-7) | 3 |
| C25 | Chemically-Induced Disorders | Percent Medication Adherence at 3-month Follow-up Assessment | 1 | Percentage of Days Used Drugs or Alcohol | 2 |
| C25 | Chemically-Induced Disorders | Clinician-assessed Depression Rating at 3 Month Follow-up Assessment | 1 | Length of Hospital Stay | 2 |
| C25 | Chemically-Induced Disorders | CD4+ Lymphocyte Count at 12-month Follow-up Assessment. | 1 | Assessment of Bioequivalence of Prototype Mini Lozenges With Nicorette Mini Lozenge by Measuring Area Under the Plasma Concentration Versus Time Curve From Time Zero to Time t (AUC [0-t]) | 2 |
| C25 | Chemically-Induced Disorders | Change in Serum 3a-androstanediol Glucuronide | 1 | Assessment of Bioequivalence of Prototype Mini Lozenges With Nicorette Mini Lozenge by Measuring Area Under the Plasma Concentration Versus Time Curve Calculated From Time Zero to Infinity (AUC [(0-inf]) | 2 |
| C25 | Chemically-Induced Disorders | Time to First Rescue-free Laxation (Following the First Dose of Study Drug). | 1 | Percent Abstinent From Tobacco at Week 32 (7 Day Point Prevalence) | 1 |
| C26 | Wounds and Injuries | Sum of Pain Intensity Difference at Rest and on Weight Bearing Over 6 Hours on Day 1 and Over 2 Hours on Day 3 | 3 | Sum of Pain Intensity Difference at Rest and on Weight Bearing Over 6 Hours on Day 1 and Over 2 Hours on Day 3 | 9 |
| C26 | Wounds and Injuries | Number of Patients With Cutaneous Bacterial Load After Surgery | 1 | Sum of Pain Intensity Difference (SPID) on Weight Bearing Over 3 Days (SPID WB0-3) | 3 |
| C26 | Wounds and Injuries | Degree of Filling of the Lesion by Repair Tissue at 12 Months Through MRI. | 1 | Sum of Ankle Pain Intensity Difference on Weight Bearing Over 24 Hours After Dose 1 (SPID WB24) | 3 |
| C26 | Wounds and Injuries | The Difference in Total Bacterial Counts Measured in Colony Forming Units (CFU) as Determined by Quantitative PCR Analysis. | 1 | Sum of Pain Intensity Difference at Rest Over 24 Hours on Day 1 (SPID R24) | 2 |
| C26 | Wounds and Injuries | Number of Inpatient Operating Room Debridements in Surgically Dehisced Wounds | 1 | Local Dynamic Stability (Knee During Turning With the Prosthesis on the Inside of the Turn) | 1 |
| F03 | Mental Disorders | Secondary Efficacy Endpoints - Vaginal Mucosa Assessment (Vaginal Color) | 12 | Vital Signs: Systolic and Diastolic Blood Pressure Levels | 12 |
| F03 | Mental Disorders | CSF IL-6, sTREM2, HMGB1, Albumin, IgG | 10 | Mean Change From Baseline (CFB) at Day 21 in Neurocognitive Function as Determined by Central Nervous System Vital Signs (CNS-VS) Test Battery | 9 |
| F03 | Mental Disorders | Percent Change From Baseline to 12 Week Endpoint in High-Density Lipoprotein Cholesterol (HDL-C) and Low-Density Lipoprotein Cholesterol (LDL-C) | 4 | Mean Change From Baseline (CFB) at Day 84 in Neurocognitive Function as Determined by Central Nervous System Vital Signs (CNS-VS) Test Battery | 9 |
| F03 | Mental Disorders | Change From Baseline to 12 Week Endpoint in Fasting Lipid Parameters Including Lipoprotein Subclasses | 4 | Incidence of Non-delirium Complications After Surgery | 8 |
| F03 | Mental Disorders | Target Engagement Assays: Change From Baseline in Filamin A (FLNA) Linkages to alpha7 Nicotinic Acetylcholine Receptor (alpha7nAChR) and Toll-like Receptor 4 (TLR4) in Subject Lymphocytes | 4 | Median Baseline and Change From Baseline in Body Mass Index (BMI) During Phase 3 | 6 |
